# Chromophobe renal cell carcinoma with liposarcomatous dedifferentiation: a case report

**DOI:** 10.11604/pamj.2022.42.206.34595

**Published:** 2022-07-14

**Authors:** Asmaa El Kebir, Evrard Niyonkuru, Mahmoud Alafifi, Nisrine Bennani Guebessi, Mehdi Karkouri, Rachid Aboutaieb

**Affiliations:** 1 Department of Pathology, Ibn Rochd University Hospital Center, Casablanca, Morocco,; 2 Faculty of Medicine and Pharmacy, Hassan II University of Casablanca, Casablanca, Morocco,; 3 Department of Urology, Ibn Rochd University Hospital Center, Casablanca, Morocco

**Keywords:** Chromophobe renal cell carcinoma, liposarcomatous dedifferentiation, Kidney neoplasm, case report

## Abstract

Chromophobe renal cell carcinoma with liposarcomatous dedifferentiation is a very extremely rare tumor and only few cases have been reported in the literature. Here we report a case of a 37-year-old woman who presented with a pain and a palpable mass in the right flank. The abdominal computed tomography (CT) scan found a renal tumor and the patient underwent a right radical nephrectomy with adrenal gland resection. After the histological examination of the specimen completed by immunohistochemical and molecular study, a diagnosis of chromophobe renal cell carcinoma with liposarcomatous dedifferentiation was made. The patient received adjuvant chemotherapy. Afterwards, she developed bone metastasis and died 13 months after the surgery. Chromophobe renal cell carcinoma with liposarcomatous dedifferentiation is a rare tumor associated with a poor prognosis and a metastatic potential.

## Introduction

Renal cell carcinoma is the common renal tumor in adults and represents approximately 3% of all adult cancers [1]. Chromophobe renal cell carcinoma is the third histological subtype of renal cell carcinoma in frequency after clear cell renal cell carcinoma and papillary renal cell carcinoma. It represents about 5.9% of all renal cell carcinoma cases and has a good prognosis but shows a poor prognosis when it is associated with sarcomatoid differentiation [2]. Chromophobe renal cell carcinoma with liposarcomatous dedifferentiation is an extremely rare tumour and to the best of our knowledge, only few cases have been reported in the literature. Herein, we present the case of chromophobe renal cell carcinoma with liposarcomatous dedifferentiation in a 37-year-old woman.

## Patient and observation

**Patient information:** a 37-year-old woman, without past medical history, presented pain and palpable mass in the right flank with alteration of overall health. The symptoms started 4 months prior to her first medical visit.

**Clinical findings:** at physical examination, there was a large and painful mass on the right flank. The rest of the examination was unremarkable.

**Diagnostic assessment:** an abdominal CT scan revealed a large heterogeneous mixed tumour of encapsulated appearance with calcifications in the right kidney. The diagnosis of renal cell carcinoma was retained according radiological features. The patient underwent a right radical nephrectomy with adrenal gland resection.

**Figure 1:**
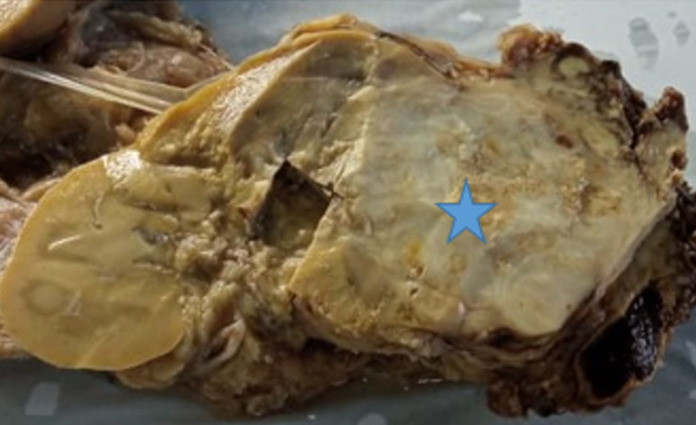
gross examination of the specimen showing a white beige neoplasm, encapsulated, with yellowish, necrotic and hemorrhagic areas (blue arrow)

**Figure 2:**
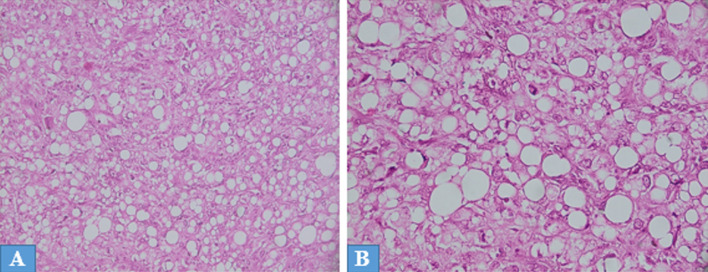
histological examination, hematoxylin and eosin stain, (A, 10x) and (B, 20x) showing the chromophobe renal cell carcinoma and high-grade sarcomatous component with sheets lipoblasts pleomorphics

**Figure 3:**
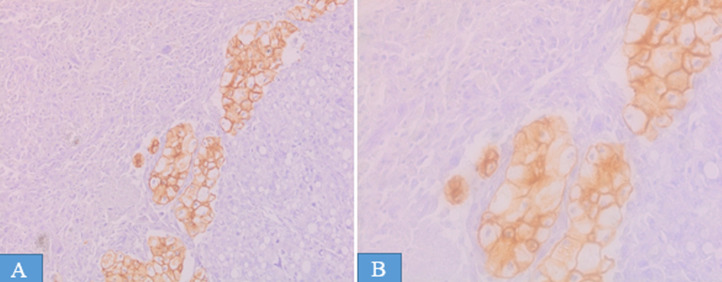
immunohistochemistry, (A, 20x) and (B, 40x); chromophobe renal cell carcinoma showing positive reactivity for cytokeratin AE1/AE3

**Diagnosis:** the gross examination of the specimen revealed a white beige neoplasm, encapsulated, measuring 19x18x17 cm, with yellowish, necrotic and hemorrhagic areas (Figure 1). There was no embolus of the renal vein or invasion of the renal capsul and ureter. No extension into perirenal fat was noted. The histopathological examination showed two distinct morphologic components, chromophobe renal cell carcinoma and sarcomatous component (Figure 2): the sarcomatous component was predominant and constituted of sheets lipoblasts pleomorphics with micro and macrovacuolated cytoplasms notching the nuclei in concave way. There were areas of undifferentiated high-grade sarcomatous composed of often large rounded cells containing hyperchromatic nuclei sometimes multinucleated with numerous mitotic figures. Chromophobe renal cell carcinoma was a minority and made of some carcinomatous clusters composed of joined large cells, with well-defined cytoplasmics borders. The cytoplasms were pale or eosinophilic containing irregular small nuclei surrounded by perinuclear haloes. These components were focally intermingled. Immunohistochemically, Chromophobe renal cell carcinoma cells were positive for cytokeratin AE1/AE3 and cytokeratin 7 (Figure 3). The sarcomatous component was focally positive for S-100 protein and was negative for cytokeratin AE1/AE3, cytokeratin 7, desmin, myogenin and mouse double minute 2 homolog (MDM2). The molecular study by fluorescence in situ hybridization technique showed an absence of amplification of MDM2. Based on the histological, molecular and immunohistochemical findings, we concluded a diagnosis of chromophobe renal cell carcinoma with liposarcomatous dedifferentiation.

**Therapeutic interventions:** after the nephrectomy, the patient received adjuvant chemotherapy.

**Follow-up and outcome of interventions:** four months after the surgery, she developed bone metastasis and was treated with radiotherapy. The patient died 13 months after the surgery.

**Informed consent:** the consent was obtained from the patient´s family.

## Discussion

Chromophobe renal cell carcinoma was first described in humans by Thoenes *et al*. in 1985 [3]. It arises from the intercalated cells of cortical segment of the collecting system [1]. All histologic subtypes of renal cell carcinoma can be associated with sarcomatoid. Differentiation and constitute about 1 5% of all renal malignant neoplasms [4]. Farrow et al were the first to describe renal cell carcinoma with sarcomatoid differentiation as a tumor exhibiting a histopathologic pattern consisting of an apparent mixture of malignant elements [5]. The mechanism of sarcomatoid transformation is not clearly known, but studies show that sarcomatoid component may originate from a common cell-of-origin, resulting in cells that lose their epithelial characteristics and gain mesenchymal characteristics through a process called epithelial-mesenchymal transition [6]. The sarcomatoid components are differents, and can be differentiated into fibrosarcoma, osteosarcoma, chondrosarcoma, liposarcoma, malignant fibrous histiocytoma, or undifferentiated sarcoma [7,8].

Renal cell carcinoma with sarcomatoid differentiation occurs in patients between 33 and 80years old with the average of 60 years [9]. The clinical presentation of renal cell carcinoma with sarcomatoid differentiation depends on the stage of disease at diagnosis.It is generally discovered in the metastatic stage. The symptoms are non-specific and can consist of flank or abdominal pain, hematuria, palpable flank mass, generalized weakness, appetite loss and weight loss [6,10]. The present patient was 37 years old with flank pain, palpable flank mass and overall health alteration. Radiologically, renal cell carcinoma with sarcomatoid differentiation is indistinguishable from usual type renal cell carcinoma [10]. Macroscopically, the tumor generally forms the encapsulated mass of variable size with a median of 10 cm and show invasive growth. The sarcomatous area displays dense and grey-white appearance with fleshy-to-fibrous cut surface [6,9,10]. Histologically, chromophobe renal cell carcinoma with sarcomatoid differentiation is defined as the coexistence of two distinct morphologic components, chromophobe cell carcinoma and sarcomatous component. The percentage of the sarcomatous component is different from case to case with low percentage in some cases while it can be extended to almost the entire areas in other cases [11]. In our case, the sarcomatous component showed a liposarcomatous dedifferentiation and constituted a large part of the tumor. Immunohistochemically, chromophobe renal cell carcinoma´s stain positively with cytokeratin AE1/AE3, cytokeratin 7, and epithelial membrane antigen (EMA) and negatively with cytokeratin 20, vimentin and CD10 [2,10]. The sarcomatous component showed negative reactivity for cytokeratins and positive reactivity for S100-protein in the 50% [12]. The differential diagnoses of liposarcomatous component finding in the kidney can be a retroperitoneal liposarcoma involving kidney, a renal metastasis from a primary soft tissue liposarcoma, a liposarcoma arising in angiomyolipoma, a primary renal liposarcoma and a lipoid urothelial carcinoma [12]. Primary renal liposarcoma is an extremely rare tumor and in our case, the presence of two distinct morphologic components allowed us to exclude it.

The treatment is a radical nephrectomy and subsequent adjuvant therapy (chemotherapy, radiotherapy, combined chemotherapy and radiotherapy, and immunotherapy) [10]. Chromophobe renal cell carcinoma with sarcomatoid differentiation is associated with a poor prognosis and a high risk of metastasis. The most common sites for distant metastasis are the lungs, bone, lymph nodes, liver and brain [6]. The five year survival rate for patients with renal cell carcinoma with sarcomatoid differentiation is ranged to 23.5-33% in various studies [6].

## Conclusion

Chromophobe renal cell carcinoma with liposarcomatous dedifferentiation is an extremely rare tumor with a very poor prognosis. An early diagnosis and rapid treatment can help to improve the patient outcome and increase the overall survival rate.
